# Physical Exercise in People with Chronic Kidney Disease—Practices and Perception of the Knowledge of Health Professionals and Physical Activity and Sport Science Professionals about Their Prescription

**DOI:** 10.3390/ijerph19020656

**Published:** 2022-01-07

**Authors:** Víctor Martínez-Majolero, Belén Urosa, Sonsoles Hernández-Sánchez

**Affiliations:** 1 Faculty of Human and Social Sciences, Universidad Pontificia Comillas, 28049 Madrid, Spain; burosa@comillas.edu; 2 Faculty of Sports Sciences, Universidad de Castilla la Mancha, 45071 Toledo, Spain; sonsoles@trainsplant.com

**Keywords:** chronic kidney disease, physical exercise, health professionals, cafyde, prescription

## Abstract

There is evidence on the need to include physical exercise as a treatment for diseases. A large number of professionals are involved in this, but it is not known how physical exercise is prescribed and which professionals are involved. This research has two objectives: (a) to find out the current practices in Spain regarding the prescription of physical exercise in patients with Chronic Kidney Disease (CKD) and (b) to analyse the perception that different health and physical activity professionals have of their knowledge to prescribe of physical exercise in the treatment of CKD. This is an empirical research with an ex post facto retrospective analysis of the information in a descriptive and correlational way. A total of 692 health and sports professionals participated. A questionnaire validated by a committee of experts was administered. Descriptive analyses were carried out and the differences in the study variables were analysed using Chi-square tests and one-factor Analysis of Variance. From the results obtained, we conclude there is a need to develop specific training programmes in the field of physical exercise for health professionals, as well as the establishment of multiprofessional teams for the prescription of physical exercise in CKD treatment, including physical exercise professionals (Cafyde).

## 1. Introduction

The incidence and prevalence of chronic kidney disease is increasing worldwide [1,2,3,4]. We can consider as risk factors for chronic kidney disease (CKD) those variables for which the literature has shown empirical evidence of their association with the disease. These factors include advanced age, family history of CKD, arterial hypertension and diabetes, among others [5]. Research studies with this type of patients show a greater probability for those individuals to suffer cardiovascular diseases [1,2,6,7], as well as a the progressive deterioration of their physical capacity [1,2,7,8]. Furthermore, the scientific literature shows that physical inactivity and poor exercise capacity is associated with a high prevalence of mortality in patients with chronic kidney disease (CKD) [9] and with a deterioration of their psychological and cognitive abilities [10]. It has been demonstrated that physical exercise has a positive effect on both overall health, specifically cardiovascular and aerobic capacity, and the quality of life in these CKD patients have been demonstrated [2,3,4,7,8,9].

The results of these investigations indicate the need to incorporate physical exercise as a component of the comprehensive treatment of these patients [11].

The prescription of physical exercise for the treatment of CKD has been included in the KDOQI (Kidney Disease Outcomes Quality Initiative) guidelines since 2001 [12], in which aerobic exercise is recommended from three to five weekly sessions with durations varying between 30 and 60 min per session at a low to moderate intensity [13,14]. Following a systematic review of the literature on the treatment of this type of patient by Quiu et al. [4] in 2017, the authors point out that not only aerobic exercises but also strength exercises are recommended. In these protocols, exercise variables, especially regarding type, intensity, volume, frequency of exercise and risk of injury, are not usually specified [15] and there is no existing consensus on which physical activity is the most beneficial for patients with CKD regarding these variables [7]. However, literature shows that nephrologists under-prescribe this type of activity as part of their treatments [3,8,11,16,17] and, in those cases where physical activity has been prescribed, the existing low levels of completion of prescribed exercises among patients [2,7,10], often attributed to their emotional barriers which makes it very difficult for them to adhere to their treatment [8]. One of the main factors for low prescription among nephrologists might be the lack of specialised training on how physical activity programmes should be included in the treatment of these patients [3,16,18,19,20].

In Spain, the prescription of a specific treatment for patients with CKD as well as it is follow-up, is carried out by the nephrologists in secundary care, although in the initial stages of the disease, the treatment’s follow-up is carried out in coordination with the primary care physician. Nephrologists are generally responsible for slowing the progression of CKD or preparing patients for renal replacement therapy, while primary care physicians are responsible for controlling or caring for CKD-related risk factors [21]. However, there is no clear distribution of responsibilities between these specialists, which sometimes leads to problems of communication and coordination between them [22], especially when patients are on dialysis or have undergone transplantation.

Although the ultimate responsibility for prescribing treatment in patients with CKD lies with nephrologists, the academic literature is full of references to treatment being coordinated by a multidisciplinary team, which specifically designs a physical exercise programme adapted to the capabilities and needs of patients, taking into account the multiple symptoms, complications and comorbidities of CKD [2,4,11,23,24,25,26,27,28].

The composition of the team is different depending on the type of professionals that exist in the different countries. In addition to nephrologists teams can also be composed by physiotherapists, nurses, cardiologists, exercise physiologists, specialised nephrologist nurses, nutritionists, occupational therapists, psychologists or social workers [11,23]. Generally in Spain, nurses and physiotherapists are the most frequent members of this type of teams, as they are considered health professionals and are present in hospital centres. However, there are some studies that point out the difficulties for both nurses [2,11,19,25,29,30,31] and physiotherapists [3] to give advice on the specific type of physical exercise recommended for patients with CKD due to their lack of specialised training on the topic. This lack of training relates not to health aspects but specifically to the type of physical activity.

In Spain there are two types of professionals, in the field of health, who may conflict over their professional competences, the physiotherapist who is a health professional and the graduate in Physical Activity and Sport Sciences (Cafyde) who is a sport professional. Although both have specialised training in physical exercise, they have different professional competences. In fact, in order to clarify the different competences of both professionals, the Professional Associations of Madrid-Spain that regulate these professions (Professional Association of Physiotherapists and Official Association of Graduates in Physical Activity and Sport Sciences), have signed a collaboration agreement on 23 December 2019 in which the different competences are clarified according to the regulations that regulate both (Order CIN/2135/2008 of 3 July [32] and in the Resolution of 18 September 2018 of the General Secretariat of Universities [33]). Considering the above, physiotherapists should be in charge of the care specific to their discipline, through treatments with physical means and agents, aimed at the recovery and rehabilitation of people with somatic dysfunctions or disabilities, as well as their prevention. They must know the physiopathology of diseases, identifying the manifestations that appear throughout the process, as well as the medical-surgical treatments, fundamentally in their physiotherapeutic and orthopaedic aspects. Conversely, the Physical Activity and Sport professional, as specified by the Spanish Ministry of Health, Consumption and Social Welfare, should be in charge of the assessment, planning, design, evaluation, development and execution of physical-sports activities; physical exercise aimed to maintain, develop, improve, optimise and recover the physical condition and coordination abilities of individual, with the objectives of improving their quality of life; as well as preventing re-educating, re-adapting and re-training those injuries and pathologies (diagnosed and/or prescribed by a doctor), by means of physical-sports activities and physical exercises adapted to their characteristics and needs.

Many authors point out that the presence of an exercise professional in these teams is essential [2,11]. Their main role would be to assess patients and design the most beneficial exercise programme. They are absolutely necessary, according to the literature, in both intra- and extra-dialysis treatments in order to adapt them to the individual needs and conditions of the patients [11,14,34], establishing the frequency, intensity, type and duration of the exercise that the physical exercise programme should include. In this sense, in Spain, the collaboration between physiotherapists and Cafyde graduates is essential.

Graduates in Cafyde in Spain are comparable to those in Sciences and Techniques of Physical and Sporting Activities in France, to professionals in Sport and Exercise Sciences in Italy, or Physiology and Sport Sciences in the United Kingdom. All of them having different professional fields in which they work, ranging from education, sports coaching, sports management or leisure and recreation to exercise for people with disabilities or physical exercise for health.

Although graduates in Physical Activity and Sport Sciences are not considered as a health profession in Spain, it would be advisable to include them in these teams as they are the professionals who are assigned the competences of “Knowing how to guide, design, apply and technically-scientifically evaluate physical exercise, physical activity and sport, taking into account their characteristics and needs, in a population with pathologies and health problems or similar that have been previously diagnosed and/or prescribed by a doctor” according to the Resolution of 18 September 2018 of the General Secretariat of Universities, which publishes the Agreement of the Council of Universities of 17 September establishing recommendations for the proposal by universities of verification reports of the official degree and which was published in the Official State Gazette on 20 September 2018 [33]. They are the only graduates with this type of competences, in contrast to the health professionals. In Spain, the prescription of treatment for chronic renal patients, including aspects of physical exercise, is the responsibility of the nephrologist, advised by the multi-professional team that he or she directs.

The Physical Activity and Sport Science professional knows which specific physical tests a person must perform to evaluate the specific aspects of their physical condition and, taking them into account, plan the training. It is the specialist with the best training to control the exercise load, who can specify the intensity, volume, duration, frequency, density and adequate recovery to achieve the greatest benefits in the body of the person, reducing the level of risk.

Considering what has been argued so far, it is evident the importance of developing physical exercise programmes by health and physical activity and sport professionals in as a part of the treatment of chronic renal patients undergoing renal replacement therapy, and that these programmes form a constituent part of the comprehensive treatment prescribed. Therefore, the physical activity and sport science professional should be part of the multidisciplinary teams, contributing their competence in the evaluation of the patient and in the design of these programmes.

It is necessary to know what practices are carried out in this respect in Spain. This will allow us to know how physical exercise is prescribed and which professionals are involved. In addition, it would be useful to know the self-perception that these professionals have on their knowledge to prescribe this type of treatment.

With this aim in mind, this research was carried out with the twofold objective of (a) finding out the current practices carried out in Spain regarding the prescription of physical exercise in CKD patients and (b) being able to analyse the perception that the different health and physical activity professionals have of their knowledge in prescribing and advising on physical exercise in the treatment of CKD.

## 2. Materials and Methods

### 2.1. Participants

In order to carry out this study, 692 health and sports professionals (Table 1) who may have a relationship with chronic kidney disease (CKD) patients requiring renal replacement therapy with haemodialysis, peritoneal dialysis or transplantation participated. Random sampling was not possible to select participants for this study. Specifically, non-probabilistic convenience sampling had to be carried out using the snowball technique, as the participants were contacted through different platforms, associations, events, professional associations, congresses, social networks, etc. To improve potential sampling biases, a large number of all types of professionals involved in chronic kidney disease were selected. Specifically, among the healthcare professionals who participated, there were 83 nephrologists, 207 nurses, 202 physiotherapists and 200 professionals from the Physical Activity and Sport Sciences (Cafyde). The inclusion criteria were to be of legal age and to be currently active in the professional field and/or working with chronic renal patients. The study was approved by the ethics committee of the Universidad Pontificia Comillas and participation was voluntary, all of them signed their consent to participate in the study. This legal consent was designed following the privacy and data protection policy of the university, which ensures the anonymous treatment and only for the purposes of this research of the data collected, in addition to the protection of the same and the right of access, rectification and deletion of them by the respondent.

### 2.2. Design and Procedures

This is an empirical research using an ex post facto retrospective analysis of the information. The objectives of the study are therefore descriptive and correlational [35].

To conduct the study, parallel forms of the same questionnaire were designed for each type of professional, adapting only two questions for Cafyde participants, which corresponded to the hospital setting. Specifically, those relating to whether there is an intra-dialysis physical exercise programme in the hospital centre and who is in charge of supervising this programme. These questions were eliminated for Cafyde participants as they are not health personnel and cannot work in the hospital setting. Prior to implementation, a validation of the content and structure of the questionnaire was carried out using the expert judgement procedure [36]. A set of 12 professionals (three nephrologists, four nurses, three physiotherapists and three physical activity and sport science professionals) with extensive professional and research experience participated. Each judge assessed the relevance, adequacy, clarity and consistency of each item using a 4-point Likert-type scale. All expert ratings were very positive for all items. For those items that scored below 3, minor modifications were made to the wording of some of the items based on the suggestions made by the experts. The final version of the questionnaire was distributed via the European Commission’s Eusurvey platform to the different professionals. This survey and its respective parallel forms were structured in two blocks, the first collecting information on the prescription of physical exercise in CKD patients and the second on the perception of the training they had for its prescription.

Regarding the first block, the different forms of the questionnaire applied to health professionals (nephrologists, nurses and physiotherapists) collected the following information: profession, inclusion of physical exercise in the treatment of people undergoing renal replacement therapy, knowledge of what type of physical activity is most recommended for this population, referral to other professionals for the prescription of physical exercise, training on the prescription of physical exercise during their studies, knowledge of the fundamental variables of training, perception of the need to receive specific training and multidisciplinary work and specific questions on in-hospital physical exercise.

In the form of the questionnaire applied to the physical activity and sport science professionals, the same information was collected as in the health professionals’ questionnaire, but the information on their job position was added, and the specific questions on in-hospital physical exercise were eliminated as they could not practise their profession in hospitals as they were not considered to be a health profession.

The second block of questions in the survey, which referred to the perception of their current knowledge and training needs in relation to the field of physical exercise, consisted of a set of Likert-type items scored between 0 and 6, 0 being the lowest value and 6 the highest and most positive value.

### 2.3. Statistical Analysis

Statistical analyses were carried out using the SPSS version 26 programme with the data collected. These analyses consisted mainly in the descriptive analysis of the different variables studied by obtaining percentages for qualitative variables as well as the mean and standard deviation statistics for quantitative variables. Likewise, an assessment of the differences in the variables on the prescription of physical exercise in treatment and the perception of physical exercise training of the different professionals were analysed by means of Pearson’s Chi-square tests for qualitative variables, where the response conditions allowed their calculation, and one-factor Analysis of Variance and Scheffé test for subsequent analyses for quantitative variables. The confidence level was 95% (*p* < 0.05). Preliminary analyses were carried out to check the application requirements for the analyses of variance.

## 3. Results

The results obtained from the first block of the survey to the questions regarding practices on the prescription of physical exercise for patients with CKD, from the different professional profiles of the participants, can be seen in Table 2.

When asked whether they were aware of the effects physical exercise had on CKD patients (dialysis and transplant), a large majority of health professionals answered affirmatively (91.6% of nephrologists, 76.3% of nurses, 71.3% of physiotherapists), although only 47.5% of physical activity and sport professionals answered in the affirmative. The Chi-square test showed a difference between the proportions of responses from professionals in favour of nephrologists and against exercise professionals. However, when asked, in the case they indicate which physical exercises CKD patients should perform, whether they control all the necessary variables of sports training (volume, intensity, density, periodicity and rest), the professionals most closely related to physical activity, physiotherapists (45.8%) and Cafyde professionals (60%) are the ones who indicate yes in a higher percentage, especially in the case of the latter. In contrast, a very small percentage of nephrologists (11.6%) and nurses (18.4%) answered affirmatively. A statistically significant difference was observed between the responses of the professionals in this aspect.

With regard to whether they specify the type of physical activity (e.g., walking, running, swimming, cycling, etc.), a high percentage of all professionals’ responded in the affirmative, although it is true that the percentage of nurses is significantly lower than the rest. They do not have homogeneous answers when it comes to identifying the physical activity that according to them is most recommendable. In general, the majority of respondents, in all professional categories, indicated physical activity that combines aerobic resistance and strength exercises, followed by only aerobic resistance activity and lastly, with a very low percentage among respondents, strength activity. It should be noted that 22.7% of nurses and 15.8% of physiotherapists did not know which physical activity is the most recommendable.

The responses of the subjects surveyed to the block of questions relating to working with other professionals, in terms of the physical exercise prescribed for CKD patients, indicated that, in general, physiotherapists and Cafydes considered the assessment of other professionals in the majority of cases (over 96%), something that occurs to a lesser extent in nurses (79%) and nephrologists (65.8%). The responses of these two groups of professionals differ significantly.

Healthcare professionals indicated that they usually work together without the presence of Cafyde professionals. A high percentage of nephrologists did not make referrals for the prescription of physical exercise (77.1%), while the rest of the professionals did, which differed significantly from the former. The reasons given for not making this referral were mostly “not knowing specialised professionals” and secondly not considering it necessary (10.3%), with nurses giving similar responses. Physiotherapists and Cafydes professionals answered in a different way, with the percentage of those who said they did not need to do referrals for the prescription of physical activity being much higher than the previous ones. In the case of referrals, nephrologists indicate that they mainly refered to physiotherapists or Cafydes professionals, nurses to physiotherapists, the latter to Cafydes professionals Cafydes among other physical activity and sport professionals.

When considering which professionals should design the specific physical exercise programme, nephrologists and nurses most frequently answered that a team of nephrologists, nurses, physiotherapists and Cafyde professionals with 31% and 25% respectively. Of this possible multidisciplinary team, physiotherapists and Cafydes professionals excluded nurses. Multidisciplinary team in health professionals being the most common response, unlike physical activity professionals who considered they are the ones who should design the programme (43%) or in collaboration with nephrologists (24%). It is noteworthy that most professionals indicated that a Cafyde professional should be present in this team.

Finally, the healthcare professionals were asked about the existence of an intra-dialysis physical exercise programme in their centre, 24.1% of nephrologists and 11.6% of nurses answered yes. Most physiotherapists did not work in the hospital setting. In the affirmative cases, it was noted that the supervisors of the programme were usually nurses and physiotherapists.

Results obtained in the second block of the survey, consisting of the questions on the training of the different professionals, can be seen in Table 3.

In this set of questions, the professionals were asked to rate their training on a scale of 0–6. It is surprising to see the results on the reduced training and competence that these professionals indicated they have received in terms of physical exercise in the case of health professionals and in health issues in the case of Cafyde professionals. We can observe the existence of significant differences between them, in the amount of training received in their university studies, with physiotherapists being the ones who thought that they have had a greater presence of these contents in their education in contrast to the rest of the professionals. Physiotherapists were also the ones who rated their training for the prescription of physical exercise in CKD patients more highly than nurses and Cafydes professionals, although they did not differ in this aspect from nephrologists. Conversely, both physiotherapists and Cafydes indicated having a higher degree of competence in specifying training variables in the prescription of physical activity when compared to what was indicated by nephrologists and nurses, although they did not differ in this respect from each other.

With regard to the importance attributed by the professionals to having adequate specific training in the effects of physical exercise for CKD, although they all indicated that it is of high importance, nephrologists were the ones who gave significantly less value to this aspect than the rest of the professionals.

Finally, all professionals, both health professionals and physical activity and sport professionals, pointed out the high need to establish this physical exercise prescription in a multidisciplinary team. Although there were significant differences in this aspect, physiotherapists and Cafydes professionals indicated that it was more necessary than nephrologists, with a statistically significant difference between them.

## 4. Discussion

Regarding the first objective of the present study: all professionals consider the effects of physical exercise in patients with renal replacement therapy in a high percentage and specify specifically what type of activity should be performed, which confirms the importance and benefits of regular physical activity in these patients [2,3,4,8,9,11,37].

However, both nephrologists and nurses do not control a high percentage of the training variables, which contradicts some articles based on the fact that physical activity should be programmed by both the nephrologist and the dialysis staff, but is congruent with the results obtained by other researchers [2,3,11,16,18,19,20,25,29,30,31].

The opposite situation occurs with Cafyde who know how to plan the physical training, but do not have specific knowledge about the effects of physical exercise in renal patients on renal replacement therapy [37]. They are also often not present in this planning teams when treatment is established.

Both nephrologists and nurses consider that the physical exercise programme should be designed jointly in a multidisciplinary team made up of nephrologists, nurses, physiotherapists and Cafyde professionals. All professionals indicate the need for the programme to be designed with the participation of Cafyde [2,11,14,34]. This type of joint work between different professionals is indicated in the specialised literature as the most suitable [2,4,11,23,24,25,26,27,28].

However, many of the professionals do not refer to other professionals since a high percentage of them do not know professionals specialised in the prescription of physical exercise in people undergoing renal replacement therapy or, on the contrary, as in the case of nephrologists, they do not consider such referral to be necessary. Although it could also be due to the fact that nephrologists and nurses have not received sufficient specific training during their university education and they show a low competence to point out some training variables.

In our study, we found that there is a low percentage of intra-dialysis physical exercise programmes in hospitals and that those responsible for such programmes, if they exist, are usually nurses or physiotherapists. This could confirm what some studies indicate that nephrologists do not consider it their responsibility to prescribe physical exercise for dialysis patients [34] or to prescribe programmes that are easily completed by the patients themselves at home [38].

In this research, there is no consensus on what type of physical activity is the most recommendable for this type of patients according to the different professionals, with mixed training and aerobic resistance training being the more predominant. Although nephrologists did not indicate that it was necessary for them to perform only strength exercises, despite the results found in the literature on their positive results with these patients [37,39]. This lack of consensus may be due to the little training received in the field of physical exercise during their university studies, as well as the lack of additional training in this area, a fact that the results of this survey corroborate. The professionals who consider themselves as the most competent in specifying specific training variables are physiotherapists and Cafyde. All the professionals surveyed indicated the importance of good specialised training in order to prescribe the physical exercise to be performed as a constituent part of CKD treatment, as well as the need to work in multidisciplinary teams. Although these last two aspects are considered to a lesser extent by nephrologists. These shortcomings in physical exercise training could be solved by creating specialised itineraries in undergraduate education or through specialised graduate programmes in physical exercise as a means of treatment in chronic kidney disease for health professionals. Similarly, in the case of Cafyde professionals, similar training would be necessary but on kidney disease.

The study has some limitations to mention. It would have been desirable to be able to select a representative sample of the different professionals through random procedures. It would also have been interesting to know the patients’ opinion on physical exercise as part of their treatment, on the barriers that may affect adherence to treatment.

## 5. Conclusions

In summary, the data found in our study allow us to conclude, on the one hand, that it would be necessary to develop specific training programmes in the field of physical exercise for healthcare professionals and kidney disease trainings programmes for physical activity and sport science professionals who deal with renal patients undergoing renal replacement therapy. These programmes would include an assessment of the importance of this type of activity for treating CKD, as well as the benefits that can be derived from it for these patients. It would also be interesting to consider a revision of the curricula of these professionals and to integrate training in physical exercise in university degrees [11].

On the other hand, it would also be interesting to consider a revision of the curricula of these professionals and to integrate training in physical exercise in university degrees [22]. On the other hand, it would be necessary to implement more physical exercise programmes in hospital dialysis centres and, in turn, to establish a specialised multidisciplinary team responsible for the prescription, supervision and individual adaptation of the physical exercise programme in patients with renal replacement therapy [11,40]. In multidisciplinary teams, each professional from different fields should have their roles well defined so that the effectiveness of the programme is maximised and should incorporate professionals from the physical activity and sports sciences in its design, as they have the greatest training in the type and fundamental variables of training.

More research is needed to provide empirical evidence on the training programmes of these professionals, the current use of physical exercise in the treatment of CKD patients, and the differences in referral patterns between different professionals. But above all, it is necessary to carry out experimental studies on what type of physical activity has the most beneficial results for these patients. Research is also needed to analyse the effectiveness of the collaborative work of these multi-professional teams, so that patients adhere to physical exercise, benefit of physical activity to a greater extent, and therefore their quality of life.

## Figures and Tables

**Table 1:** Distribution of study participants according to their profession.

Professión	N	%
Nephrologist	83	12
Nurses	207	30
Physiotherapists	202	29
Cafyde	200	29
Total	692	100

**Table 2:** Descriptive values and Chi-square test results for qualitative variables on physical exercise in CKD treatment.

Variables	Nephrologists	Nurses	Physiotherapists	Cafyde	Analysis
It takes into account the effects of physical exercise	Yes 91.6%	Yes 76.3%	Yes 71.3%	Yes 47.5%	Chi = 67.77
	No 8.4%	No 23.7%	No 28.7%	No 52.5%	*p* < 0.00001
Control all variables of sports training	Yes 11.8%	Yes 18.4%	Yes 45.8%	Yes 60%	Chi = 151.08
	No 46.1%	No 31%	No 7.6%	No 6.3%	*p* < 0.00001
	Some 42.1%	Some 50.6%	Some 46.5%	Some 33.7%	
Specify the specific physical activity	Yes 82.9%	Yes 77.8%	Yes 85.4%	Yes 88.4%	Chi = 9.3242
	No 17.1%	No 22.1%	No 14.6%	No 11.6%	*p* < 0.05
Most recommended physical activity	Mixed 50.6%	Mixed 49.3%	Mixed 69.3%	Mixed 62%	
	R. Aerobic 36.1%	R. Aerobic 21.7%	R. Aerobic 12.9%	R. Aerobic 6%	
	Strength 0%	Strength 3.4%	Strength 1.5%	Strength 5%	
	No type of physical activity 2.4%	They do not know what physical activity 22.7%	Unknown act. physical 15.8%	No type of act. physical 0.5%	
		No type of physical activity 1.9%			
It takes into account the assessment of other professionals	Yes 65.8%	Yes 79.1%	Yes 96.5 %%	Yes 96.8%	Chi = 80.68
	No 34.2%	No 20.9%	No 3.5%	No 3.2%	*p* < 0.00001
Professionals who work together to indicate physical exercise	Nurses and physiotherapists 16%	Nephrologists 28.8%	Nephrologists 13.7%	Nephrologists 13%	
	Nurses 12%	Nephrologists and nurses 16%	Physiotherapist nephrologists and cafyde 13.7%	Nephrologists, nurses, physiotherapists and cafyde 12%	
	Nephrologists and nurses 10%	Nephrologists and physiotherapists 13.6%	Nephrologists, nurses, physiotherapists and cafyde 7.9%		
Refer to other professionals for the prescription of physical exercise	Yes 18.1%	Yes 62.3%	Yes 69.8%	Yes 69%	Chi = 117.30
	No 77.1%	No 34.3%	No 23.3%	No 17.5%	*p* < 0.00001
	Not necessary 4.8%	Not necessary 3.4%	Not necessary 6.9%	Not necessary 13.5%	
Reason for not referring to other professionals for the prescription of physical exercise	They do not know specialized professionals 77.9%	They do not know specialized professionals 76.9%	They do not know specialized professionals 45.9%	Do not know specialized professionals 61.3%	
	Not necessary 10.3%	Not necessary 5.1%	Not necessary 42.6%	Not necessary 33.9%	
	They don’t need 4.4%	They don’t need 2.6%	They don’t need 9.8%	They don’t need 4.9%	
Which professionals do you refer to for the prescription of physical exercise?	Physiotherapists 33%	Physiotherapists 26.4%	Cafyde 22.7%	Other coffee 37.7%	
	Physiotherapists and cafyde 20%	Nephrologists and physiotherapists 19.4%	Physiotherapist and cafyde 14.2%	Nephrologists 15.9%	
	Cafyde 20%	Cafyde 13.2%	Nephrologists 12.1%	Nephrologists and cafyde 8.7%	
Professional/s who must design the specific physical exercise program	Nephrologists, nurses, physiotherapists and cafes of 31.7%	Nephrologists, nurses, physiotherapists and cafyde 25.1%	Nephrologists, physiotherapists and cafyde 25.2%	Cafyde 43%	
	Cafyde 17.1%	Nephrologists, nurses and physiotherapists 15.9%	Physiotherapist 15.3%	Nephrologists and cafyde 24%	
	Physiotherapist 11%	Physiotherapist 11.1%	Physiotherapist and cafyde 13.9%	Nephrologists, physiotherapists and cafyde 14%	
There is an intradialysis physical exercise program	Yes 24.1%	Yes 11.6%	Yes 3.5%	They are not professionals in the health field	Chi = 217.101
	No 67.1%	No 68.1%	No 14.4%		*p* < 0.00001
	Does not work in hospital 8.4%	Does not work in hospital 20.3%	Does not work in hospital 82.2%		
Professional/s in charge of supervising the intradialysis physical exercise program	Nurse and physiotherapist 20%	Nurse and physiotherapist 33.3%	Physiotherapists 42.9%	They are not professionals in the health field	
	Cafyde 15%	Nurse 12.5	Doctor and physiotherapist 28.6%		
		Physiotherapist 12.5%	Physiotherapist and cafyde 14.3%		

**Table 3:** Results of the Analysis of Variance and the subsequent Schefeé test of the variables on the perception of the professionals with respect to their training. (N = Nephrologist; E = Nurse; F = Physiotherapist and C = Cafyde).

	Nephrologist		Nurse		Physiotherapist		Cafyde		F	*p*	Eta2	Scheffé
	(N = 83)		(N = 207)		(N = 202)		(N = 200)					
Variables	M	DT	M	DT	M	DT	M	DT				
Amount Training in physical exercise in their university studies	0.87	1.25	1.00	1.43	1.51	1.63	1.03	1.30	6.62	<0.001	0.028	F > N;
												F > E;
												F > C
Assessment of their training to prescribe physical exercise.	2.51	1.26	2.18	1.60	2.64	1.70	2.02	1.67	5.85	<0.001	0.025	F > E;
												F > C
Perception of your competence to specify training variables	1.94	1.33	2.12	1.58	2.86	1.82	2.60	1.77	9.73	<0.001	0.041	F > N;
												F > E;
												C > N;
												C > E
Perception of the importance of having specific training in the effects of physical exercise	4.65	1.14	5.13	1.06	5.10	1.22	5.19	1.09	4.73	<0.05	0.020	E > N;
												F > N;
												C > N
Perception that the prescription of physical exercise should be a multidisciplinary work	5.18	1.12	5.49	0.81	5.54	0.87	5.54	0.93	3.57	<0.05	0.015	F > N;
												C > N

## Data Availability

Not applicable.
